# Maintaining Integrity of Worldwide FINGER Clinical Trials During the COVID-19 Pandemic

**DOI:** 10.1093/geroni/igab046.262

**Published:** 2021-12-17

**Authors:** Susanne Roehr

**Affiliations:** Institute of Social Medicine, Occupational Health and Public Health (ISAP), Leipzig, Sachsen, Germany

## Abstract

The COVID-19 pandemic presents challenges to the conduct of randomized clinical trials of lifestyle interventions. World-Wide FINGERS international network convened a forum for researchers to discuss statistical design and analysis issues they faced during the pandemic. We report experiences of three trials that, at various stages of conduct, altered designs and analysis plans to navigate these issues. We provide recommendations for future trials to consider as they develop and launch behavioral intervention trials. The pandemic led researchers to change recruitment plans, interrupt timelines for assessments and intervention delivery, and move to remote intervention and assessments protocols. The necessity of these changes add emphasis to the importance, in study design and analysis, of intention to treat approaches, flexibility, within site stratification, interim power projections, and sensitivity analyses. Robust approaches to study design and analysis are critical to negotiate issues related to the intervention.

